# The m^6^A demethylases FTO and ALKBH5 aggravate the malignant progression of nasopharyngeal carcinoma by coregulating ARHGAP35

**DOI:** 10.1038/s41420-024-01810-0

**Published:** 2024-01-23

**Authors:** Zhiyuan Yang, Siyu Zhang, Jiayan Xiong, Tian Xia, Rui Zhu, Mengyu Miao, Keying Li, Wenyue Chen, Lin Zhang, Yiwen You, Bo You

**Affiliations:** 1 Department of Otolaryngology Head and Neck Surgery, Affiliated Hospital of Nantong University, Medical School of Nantong University, Nantong, China; 2 Institute of Otolaryngology Head and Neck Surgery, Affiliated Hospital of Nantong University, Medical School of Nantong University, Nantong, China; 3 Medical School of Nantong University, Nantong, China; 4 Haimen People’s Hospital, Nantong, China

**Keywords:** Head and neck cancer, Cancer genomics

## Abstract

*N*^6^-methyladenosine (m^6^A) is an RNA modification that can be removed by demethylases [fat mass and obesity-associated protein (FTO) and AlkB homolog 5 (ALKBH5)], which regulate gene expression and cell function. We show that m^6^A levels and m6A demethylase levels are altered in nasopharyngeal carcinoma (NPC) tissues vs. normal tissues. High FTO and ALKBH5 predict a poor prognosis in NPC patients. Silencing FTO and ALKBH5 inhibited the malignant behavior of patient-derived NPC cells in a short time. However, as time progressed, the inhibitory effect of FTO or ALKBH5 was weakened, and the cosilencing of FTO and ALKBH5 maintained a better inhibitory effect. Combined transcriptome and m^6^A-seq analysis revealed a downstream target gene that was jointly regulated by FTO and ALKBH5 in NPC, and ARHGAP35 was chosen to do further study. The synergistic silencing of FTO and ALKBH5 increased the methylation level on the mRNA CDS of a new transcription factor (ARHGAP35) and positively regulate the protein coding capacity and mRNA stability of ARHGAP35, thus leading to increased expression of ARHGAP35 and inhibition of the malignant phenotype of tumor cells. Our study revealed that the growth and metastasis of NPC can be stably inhibited through synergistic silencing of the demethylases FTO and ALKBH5, which play a positive role in the treatment of NPC by regulating the downstream transcript ARHGAP35 and increasing its m^6^A level.

## Introduction

Nasopharyngeal carcinoma (NPC) is the most common primary malignancy among head and neck squamous cell carcinomas (HNSCCs) and is highly aggressive and metastatic. Even with a combination of conventional radiation, surgery and other treatments, relapse is inevitable [1]. Previous studies at our institute have mainly focused on the characteristics of explosive growth after tumor dormancy, which can affect the malignant progression of tumors by promoting angiogenesis and regulating autophagy [2, 3]. However, research on other aspects, including m^6^A modification in NPC, is still limited. Therefore, studying the mechanism of NPC cell proliferation and metastasis is helpful to find new targets and provide assistance for the application of clinical targeted therapy.

m^6^A has been identified as the most common internal modification of mRNA in eukaryotes [4]. m^6^A modification can be assembled by methyltransferases such as METTL3/14 and erased by the demethylases FTO/ALKBH5; in addition, the m^6^A recognition proteins [YTH domain family proteins (YTHDCs and YTHDFs) and insulin-like growth factor 2 mRNA binding proteins (IGF2BPs)] can also affect m^6^A levels [5–7]. m^6^A can affect cell transcription, translation and posttranslational modification by maintaining mRNA stability [8–10]. According to existing research on m^6^A modification, in leukemia cells, the expression of phosphofructokinase platelets (PFKP) and lactate dehydrogenase B (LDHB) (two key glycolytic genes) was increased through the mediation of FTO/m^6^A/YTH m^6^A RNA binding protein 2 (YTHDF2), thus promoting aerobic glycolysis in leukemia cells and significantly inhibiting the progression of leukemia [11]. FTO promotes the proliferation and migration of HNSCCs by increasing CTNNB1 expression [12]. ALKBH5 inhibits the expression of RIG-I and interferon α induced by the IKKε/TBK1/IRF3 pathway to promote HNSCC progression [13]. The above studies confirmed the role of FTO/ALKBH5 in HNSCCs. NPC is a vital HNSCC, and NPC studies on m^6^A modification have emerged. m^6^A promotes tumor resistance by enhancing the RNA stability of TRIM11 in NPC cells [14]. By maintaining the stability and expression of carcinogenic coding and noncoding genes in NPC, m^6^A modification can inhibit autophagy [14, 15]; induce EMT [16], facilitate tumor growth and metastasis [17, 18], and ultimately lead to a poor prognosis in NPC patients. A recent study revealed that FTO is significantly upregulated in drug-resistant NPC tissues and cells and leads to radiotherapy resistance in NPC by promoting the iron resistance of OTUB1 [19]. However, ALKBH5’s role in NPC has yet to be clarified. The specific mechanism and target genes of m^6^A modification in NPC are also unclear.

Rho GTPase activating protein 35 (ARHGAP35, also known as P190A) is a 172 kDa widely expressed protein that is mainly located in the intracellular nuclear body [20]. ARHGAP35 is involved in many important physiological processes, including GTPase activation, migration, cohesion and cell cycle progression [21]. Its function mostly occurs through the inactivation of RhoA. The expression of ARHGAP35 was significantly decreased in gastric cancer, colorectal cancer, lung adenocarcinoma, melanoma, endometrial cancer, glioblastoma and other malignant tumors [22–28]. The low expression of ARHGAP35 is also related to a poor prognosis in tumor patients. However, the mechanism by which ARHGAP35 regulates tumorigenesis and its role in NPC are still largely unknown.

In this study, we found that FTO and ALKBH5 expression was higher in the tissues of patients with advanced NPC, and this phenotype indicated a poor prognosis. Through preliminary in vivo and in vitro experiments, we found that silencing FTO or ALKBH5 alone could inhibit the malignant biological behavior of NPC cells for a short time, which not only verified the conclusion of the above study [19] but also revealed that ALKBH5, as another demethylase, plays a role similar to that of FTO. Moreover, over time, the inhibitory effect of silencing FTO/ALKBH5 alone on NPC cells was relatively weakened and even eventually lost. However, NPC cells with silencing of FTO and ALKBH5 can maintain a stable inhibitory effect for a long time. Therefore, we wanted to find a stable downstream molecule that is regulated jointly by FTO and ALKBH5. By high-throughput m^6^A-seq and transcriptome sequencing, we found that ARHGAP35 (p190A) could be used as a synergistic downstream target of FTO and ALKBH5. Synergistic knockdown of FTO and ALKBH5 in NPC cells increased the m^6^A level on ARHGAP35 mRNA and regulated the mRNA stability and translation of ARHGAP35. ARHGAP35 could reverse the carcinogenic effect of FTO and ALKBH5 on NPC cells. These findings reveal the important regulatory role of the FTO/ALKBH5-ARHGAP35 signaling axis. This study is the first to explore the malignant biological effects of the combined action of demethylase FTO and ALKBH5 in nasopharyngeal carcinoma. The identification of more precise targets in epigenetics can have a significant impact on the diagnosis and treatment of NPC, making this research crucial for providing new ideas in the field.

## Results

### Expression and clinical significance of m^6^A and its demethylase in NPC samples

**Fig. 1:**
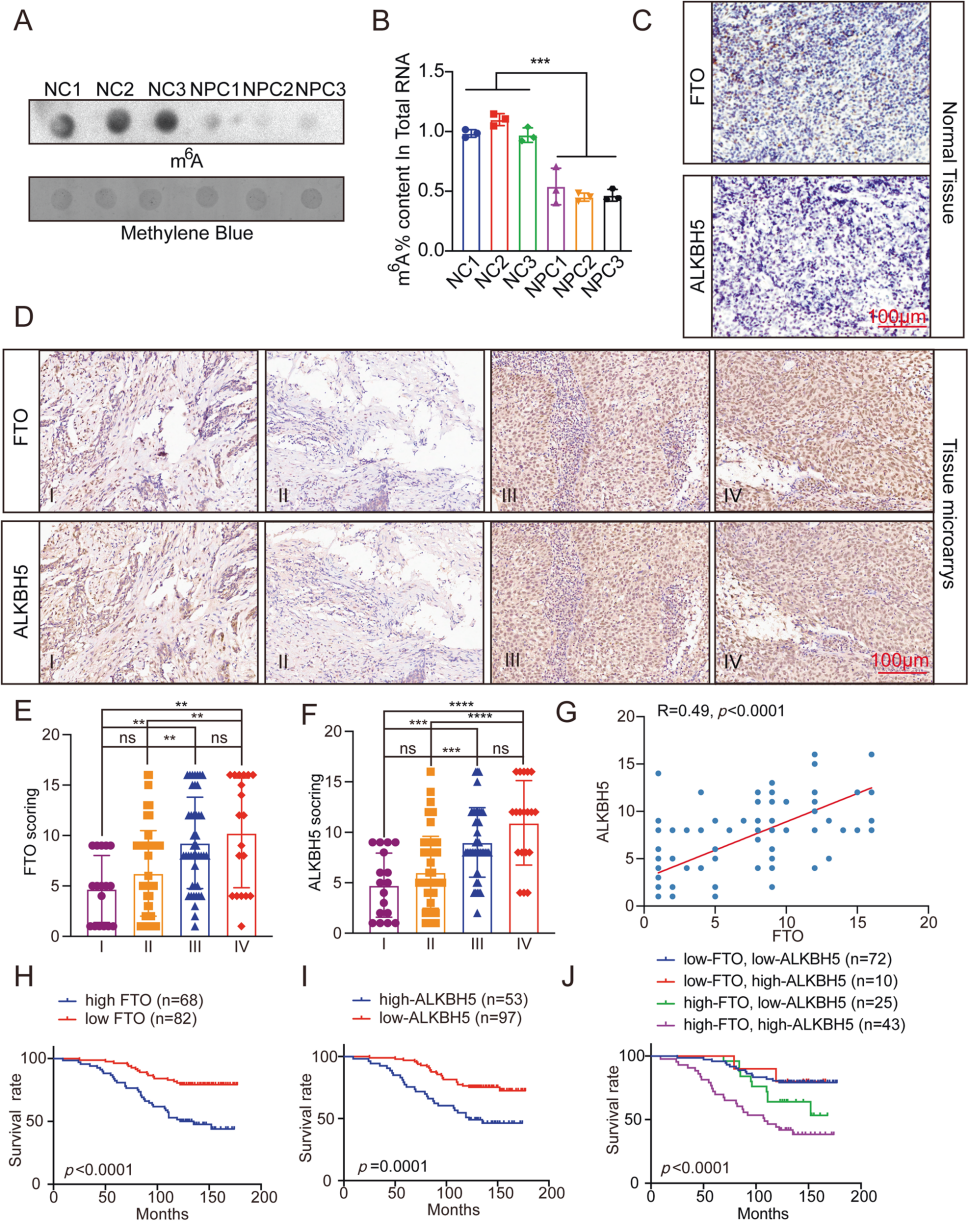
Expression and clinical significance of m^6^A and its demethylase in NPC. **A** The representative images of m^6^A dot blot detection showed the overall abundance of m^6^A in NPC patients and normal human tissues. (Methylene blue staining: as the control group. NC: normal people, NPC: NPC patients, *n* = 3) **B** The content of m^6^A in total RNA of NPC patients and normal people (the same as **A**). **C** IHC staining of representative FTO and ALKBH5 of normal tissue, scale: 100 μm. **D** IHC staining of representative FTO and ALKBH5 of NPC tissue microarray, scale: 100 μm. **E**, **F** The expression of FTO and ALKBH5 in different clinical stages were statistically compared. Single factor analysis of variance. **G** Spearman correlation analysis of FTO score and ALKBH5 score. **H**–**J** Kaplan-Meier analysis then used log-rank test to compare the overall survival rate. All experiments were performed with 3 independent repetitions. All figures show the average ± SEM of at least three independent experiments. *p < 0.05, **p < 0.01, ***p < 0.001, ****p < 0.0001.

As one of the most widely studied RNA modifications, m^6^A modification, and its related regulatory genes play important roles in the tumorigenesis and metastasis of NPC [29]. Although the current study revealed that m^6^A contributes to NPC progression by affecting RNA stability and translation of related carcinogenic target molecules [15, 18, 30], the level of m^6^A in NPC tissues and cells has not been reported. Therefore, to explore the content of m^6^A in NPC tissues, we extracted RNA from tissues from clinically diagnosed NPC patients and found that compared with that in normal tissues, the m^6^A content in NPC tissue samples was significantly reduced (Fig. 1A, B).

m^6^A demethylases are known to reduce m^6^A content. A recent study on m^6^A in NPC revealed that FTO was highly expressed in radiotherapy (RT)-resistant NPC tissues and cells [19]. Unfortunately, it did not address the expression of another demethylase, ALKBH5, in NPC. Moreover, the role of FTO/ALKBH5 in the malignant progression of NPC is not yet clear. The above conclusions have confirmed that the overall level of m^6^A is relatively low in NPC tissues. Based on our findings on the role of ALKBH5 in m^6^A modification, we hypothesized that ALKBH5 may have a similar role to FTO, and we found that the expression of the two demethylases was higher in NPC tissues than in normal nasopharynx tissues (Fig. 1C–D). Therefore, to further explore the clinical significance of m^6^A demethylase in NPC, a tissue microarray composed of samples from 150 newly diagnosed NPC patients was constructed for immunostaining, expression analysis and follow-up study of FTO and ALKBH5. The results showed that the expression of FTO and ALKBH5 was higher in NPC patients with tumor node metastasis (TNM) stages III and IV than in NPC patients with TNM stages I and II (Fig. 1D–F). This suggests that the expression of FTO and ALKBH5 may be positively correlated with the malignant degree of NPC, and we found that FTO and ALKBH5 were positively correlated (Fig. 1G). In addition, the Kaplan‒Meier survival curve analysis showed that NPC patients with high expression of FTO and ALKBH5 had a poor prognosis (Fig. 1H, I). Moreover, we analyzed the effects of these two demethylases on the prognosis of NPC patients in detail through Kaplan‒Meier survival curves: we found that the prognosis of NPC patients with high expression of both FTO and ALKBH5 was much worse than that of patients with low expression of both FTO and ALKBH5 (Fig. 1J).

The above data indicate that the overall content of m^6^A in NPC tissues is low, while high expression of its demethylase in NPC tissues predicts a poor prognosis in patients.

### The synergistic silencing of demethylases can inhibit the malignant progression of NPC more effectively than silencing of either demethylase alone

To investigate the role of the m^6^A modification demethylases FTO and ALKBH5 in the malignant progression of NPC, we first silenced FTO and ALKBH5 by constructing short hairpin RNA (shFTO, shALKBH5) (Figure S1A–H).

Next, we studied the effects of FTO and ALKBH5 knockdown on NPC proliferation and migration. Through an EdU cell proliferation experiment, we found that compared with the cells without any treatment and shCtrl cells on Day 0, after shFTO combined with ALKBH5 (hereafter referred to collectively as shFTO+shALKBH5) in NPC cells, the proportion of cells in the proliferating state was reduced to varying degrees. In addition, coknockdown of FTO and ALKBH5 had a more significant inhibitory effect on proliferation than knockdown of either factor alone. In addition, over time, the inhibitory proliferation effect of FTO and ALKBH5 knockdown alone on NPC cells was weakened or even disappeared, while coknockdown cells maintained a good inhibitory effect (Figure S2A–D). Transwell experiments showed that the shFTO+shALKBH5 group could stably inhibit the migration ability of NPC cells for a long time. However, the inhibition effect of single knockdown (shFTO, shALKBH5) on migration ability was not as good as that of coknockdown (shFTO+shALKBH5) over a short period of time, and with the extension of time, the inhibition of cell migration ability was lost (Figure S2E–H). At the final moment (around the tenth day), we collected cells for flow cytometry analysis. The results indicated that the shFTO+shALKBH5 group maintained a good proapoptotic ability compared to the single knockdown group (Figure S1I).

**Fig. 2:**
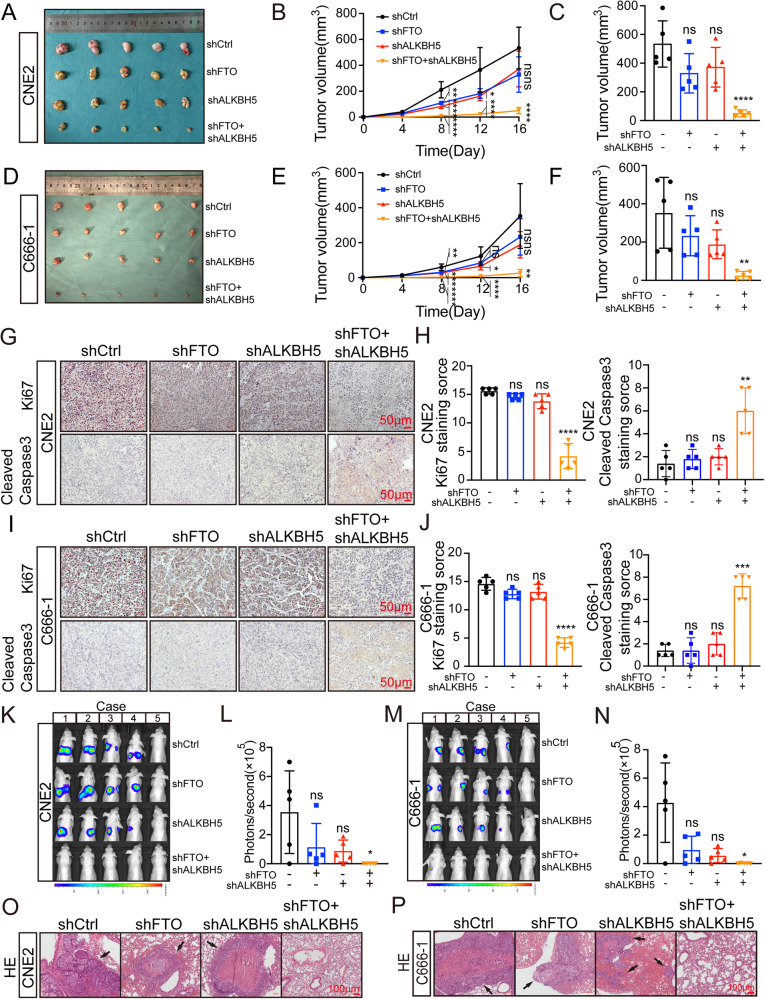
Low expression of FTO or ALKBH5 synergistically inhibits the progression of NPC in vivo. **A**–**F** The stably silenced cells of shFTO, shALKBH5, shFTO+shALKBH5 or control group of CNE2, C666-1 cells were transplanted subcutaneously into nude mice (*n* = 5 for each group). On the 16th day, the subcutaneous tumor volume was displayed. The graft volume was measured every 4 days after the tumor was transplanted subcutaneously in nude mice, and the final volume of the removed graft was measured. Single factor analysis of variance **p < 0.01, ****p < 0.0001, one-way ANOVA. **G**–**J** immunohistochemistry (IHC) was used to analyze the expression of Ki67 and Cleaned Caspase3 among tumor groups. Scale: 50 μm. (****p < 0.001, one-way ANOVA) **K**–**N** The visual and quantitative analysis of lung metastasis in BALB/c mice. by one-way ANOVA. (*p < 0.05, one-way ANOVA). **O**, **P** Representative image of lung tissue stained with HE. Scale: 100 μm.

Based on the in vitro results of the proliferation and migration of CNE2 and C666-1 cells in the shFTO, shALKBH5, and shFTO+shALKBH5 groups, we established a normal nude mouse NPC model to explore the effects of these groups in vivo. We randomly assigned 4-week-old nude mice to groups. Tumor volume was measured on the 4^th^, 8^th^, 12^th^, and 16^th^ days after subcutaneous tumor formation in the shCtrl group (Fig. 2B, E). The volume was measured after the xenograft was removed on the 16^th^ day. We found that the xenografts formed from the shFTO/shALKBH5 and shFTO+shALKBH5 groups had different degrees of growth inhibition. On the 16^th^ day, part of the xenograft volume of the shFTO/shALKBH5 group was almost the same as that of the shCtrl group, and only the shFTO+shALKBH5 group still maintained a stable inhibitory effect on tumor growth (Fig. 2A–F). In addition, immunohistochemistry of the paraffin sections of the primary tumor of each nude mouse showed that the Ki67 staining of the xenograft tumors generated from the cells of the shCtrl group was strongly positive, and the Ki67 staining intensity of the two single knockdown groups was not significantly different from that of the shCtrl group. However, the Ki67 staining intensity of the shFTO+shALKBH5 group was relatively low. Moreover, the Cleaned Caspase3 (which reports the activated form of apoptosis) staining showed an opposite trend (Fig. 2G–J). The results indicate that the combination of shFTO and shALKBH5 has a synergistic inhibitory effect on the proliferation of NPC by continuously promoting apoptosis.

Similarly, 20 nude mice were randomly assigned to the groups described above, and 1 × 10^6^ fluorescently labeled CNE2 and C666-1 cells were injected into the tail vein of each nude mouse. One month later, mice were anesthetized with sevoflurane, and imaging experiments were performed in vivo. The results showed that the lung bioluminescence intensity of some nude mice in the shFTO/shALKBH5 group was not weaker than that in the shCtrl group. However, no obvious cell bioluminescence was observed in the lungs of most nude mice in the shFTO+shALKBH5 group, and only one nude mouse had extremely weak bioluminescence in the lung (Fig. 2K–N). After in vivo imaging, nude mice injected with tumor cells in the tail vein were killed immediately, and lung metastases were observed, followed by paraffin sections and HE staining of lung tissues. One representative visual field was randomly selected from each of the 4 groups of lung tissue, and the site of metastasis is indicated by arrows. By observing HE images, we reached a conclusion consistent with in vivo imaging (Fig. 2O, P).

In summary, these experiments in vivo showed that although knockdown of FTO/ALKBH5 alone or in combination could inhibit the malignant phenotype of NPC for a period of time, the inhibitory effect of knockdown of a single demethylase on NPC could be weakened or even disappear with the extension of time. However, coknockdown of FTO and ALKBH5 can stably inhibit NPC malignant behavior.

### Analysis of NPC regulation by common downstream targets of FTO and ALKBH5

**Fig. 3:**
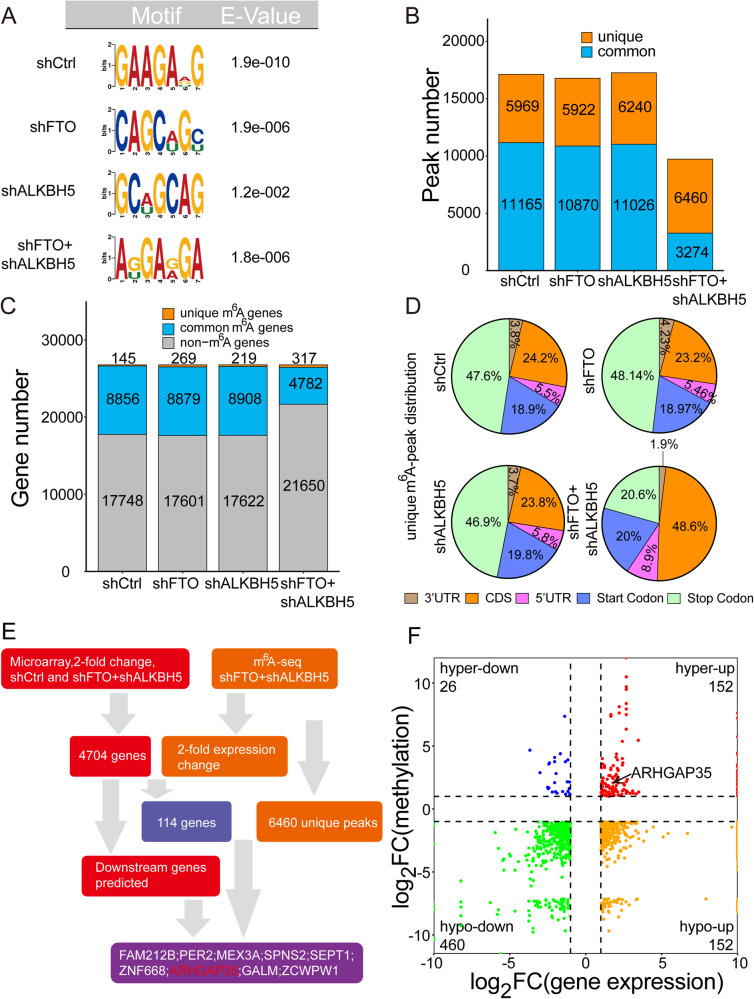
Characteristics of m^6^A modification and gene expression changes in FTO and ALKBH5 deficient cells. **A** There is a top-level consistent motif with m^6^A seq peak in CNE2 cells of shFTO, shALKBH5, shFTO+shALKBH5. **B** The number of m^6^A peaks found in m^6^A-seq in CNE2 cells of shCtrl, shFTO, shALKBH5 and shFTO+shALKBH5. **C** The number of m^6^A-modified genes identified in m^6^A-seq. **D** The distribution map of m^6^A peaks shows the proportion of m^6^A peaks specific to the designated area in shCtrl, shFTO, shALKBH5, shFTO+shALKBH5 cells. **E** Downstream analysis diagram of FTO and ALKBH5 joint regulation. **F** Positioning of the downstream of the joint regulation of FTO and ALKBH5 in the 1/4 quadrant diagram.

To verify whether the above changes in the NPC malignant phenotype were caused by the cooperative “demethylation” of m^6^A-mediated gene expression by FTO and ALKBH5, we compared the distribution of m^6^A in the shCtrl, shFTO, shALKBH5 and shFTO+shALKBH5 groups to explore the relationship between them. Using m^6^A-seq technology, we successfully located the m^6^A methylation site in shCtrl, shFTO, shALKBH5 and shFTO+shALKBH5 cells and performed accurate biological replication. The results showed that the GGACU motif was highly enriched in the m^6^A site of NPC cells (Fig. 3A).

In general, through m^6^A-seq, 17134, 16792, 17266 and 9734 m^6^A peaks were identified in shCtrl, shFTO, shALKBH5 and shFTO+shALKBH5 cells from 9001, 9148, 9127 and 5099 m^6^A-modified transcripts, respectively (Fig. 3B, C). After stable knockdown of FTO and ALKBH5 with shRNA in NPC cells, 6460 new peaks appeared in the shFTO+shALKBH5 group, of which 5969 peaks disappeared. The other 3274 peaks were found in the shFTO, shALKBH5, and shCtrl groups (Fig. 3B). Since FTO and ALKBH5 are m^6^A-modified demethylases, it is expected that these 6460 unique peaks will contain the real base targets for the joint regulation of FTO and ALKBH5. We further studied the distribution pattern of m^6^A peaks. When mRNA species were divided into the 5’ untranslated region (5’UTR), coding DNA sequence (CDS), 3’ untranslated region (3’UTR), promoter and terminal domain, we found that there were similar m^6^A distribution patterns in shCtrl, shFTO and shALKBH5 cells (Fig. 3D). Interestingly, 5969 missing peaks showed the same distribution as the total peaks, compared with 6460 unique peaks depending on FTO and ALKBH5 showing an obvious pattern, in which there is a relatively increased m^6^A deposition in CDS and 5’UTR (Fig. 3D). The m^6^A peaks in these unique mRNAs included 575 peaks in the 5’UTR, 3140 peaks in the CDS, 123 peaks in the 3’UTR, 1292 peaks in the promoter and 1330 peaks in the terminal domain.

Therefore, we wanted to determine whether these peaks were related to differentially expressed genes in the microarray analysis. In 4707 differentially regulated transcripts identified by microarray analysis, 151 genes were found in the RNA-seq dataset (m^6^A-seq input library), and 114 of these genes showed more than a 2-fold change in expression in the same direction as FTO and ALKBH5. Next, 6460 unique m^6^A peaks were screened in 114 genes with over 2-fold expression change, and 9 genes including peak sites were screened: FAM212B, PER2, MEX3A, SPNS2, SEPT1, ZNF668, ARHGAP35, GALM and ZCWPW1 (Fig. 3E). Then, we selected a possible tumor suppressor gene, ARHGAP35, from the nine candidate genes based on the previous conclusion that the combined knockdown of FTO and ALKBH5 can inhibit the malignant phenotype of NPC (Fig. 3E). From the 1/4 quadrant diagram, we found that the total mRNA expression and m^6^A levels of ARHGAP35 were significantly increased in the coknockdown of FTO and ALKBH5 group compared with the shCtrl group (Fig. 3F).

Therefore, based on the above inference results, our subsequent research will focus on this potentially important target gene.

### Relationship and clinical significance between ARHGAP35, FTO and ALKBH5

**Fig. 4:**
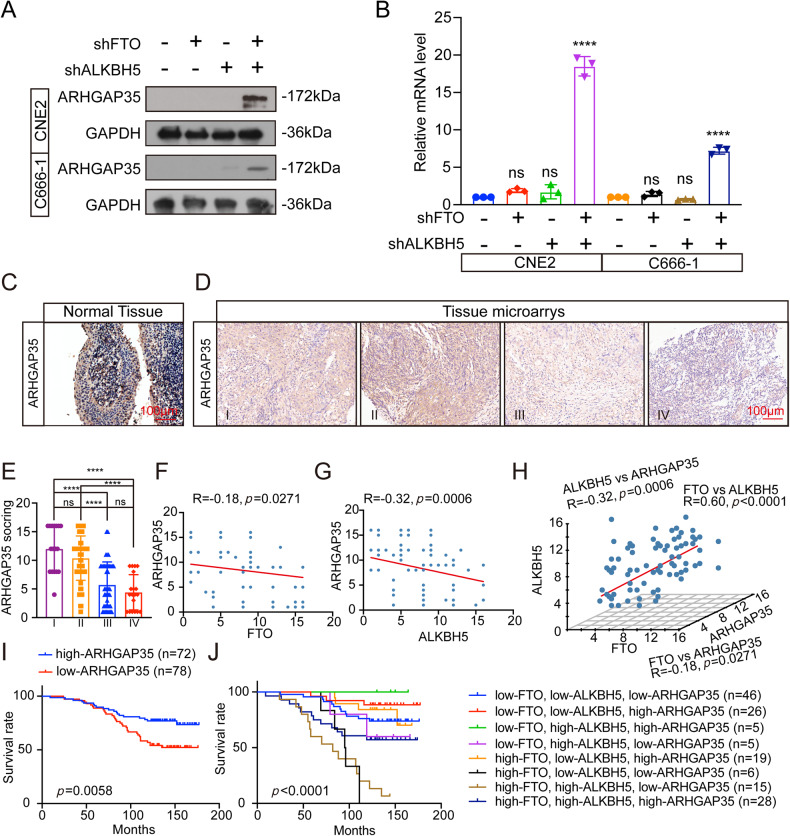
The synergistic down-regulation of FTO and ALKBH5 affects the expression and clinical significance of ARHGAP35. **A** Western blot of ARHGAP35 protein in CNE2 and C666-1 knockdown by FTO and ALKBH5 respectively or jointly. **B** RT-qPCR was used to analyze the expression of ARHGAP35 mRNA in CNE2 and C666-1 before and after FTO and ALKBH5 were respectively or jointly knocked down. The sample was normalized to GAPDH mRNA. one-way ANOVA, ****p < 0.0001. **C** IHC staining of representative ARHGAP35 of normal tissue, scale: 100 μm. **D** IHC staining of representative ARHGAP35 of NPC tissue microarray, scale: 100 μm. **E** The expression of ARHGAP35 in different clinical stages was statistically compared. Single factor analysis of variance. **F**, **G** Spearman correlation analysis of FTO score and ARHGAP35 score, ALKBH5 score and ARHGAP35 score. **H** Spearman correlation analysis of FTO score, ALKBH5 score and ARHGAP35 score in xyz-three-dimensional coordinate system. **I** The Kaplan-Meier survival time of NPC patients with ARHGAP35 was analyzed. **J** The Kaplan-Meier survival with differential gene expression of the three biomarkers was assessed. The scale bar in the images represents 100μm. Mean ± SD, ****p < 0.0001, one-way ANOVA.

To verify the predicted target molecules, western blotting and real-time quantitative PCR (qRT‒PCR) analysis were performed and revealed that compared with shCtrl cells, CNE2 and C666-1 cells with coknockdown of FTO and ALKBH5 had significantly increased expression of ARHGAP35, while cells with knockdown of FTO or ALKBH5 alone had low or even no expression of ARHGAP35 compared with shCtrl cells (Fig. 4A, B).

To further explore the clinical significance of ARHGAP35, a tissue microarray (*n* = 150) composed of the same batch of NPC patient tissues used in the previous analysis of FTO and ALKBH5 was adopted. Immunostaining and expression analysis of ARHGAP35 were performed on the tissue microarray. In addition, the expression of ARHGAP35 was also higher in normal nasopharynx tissues than NPC tissues (Fig. 4C). The results showed that the expression of ARHGAP35 was significantly higher in NPC patients in clinical stage I and II than in NPC patients in clinical stage III and IV (Fig. 4D, E). This suggests that low expression of ARHGAP35 may indicate that NPC patients are in the middle and late clinical stages. In addition, we compared the staining scores of ARHGAP35, FTO and ALKBH5 in the tissue microarray and found that ARHGAP35 was negatively correlated with FTO or ALKBH5 (Fig. 4F, G). Moreover, we further constructed the xyz three-dimensional coordinate system, from which the relationship between ARHGAP35, ALKBH5 and FTO can be more intuitively observed (Fig. 4H). Moreover, Kaplan‒Meier survival curves showed that NPC patients with higher ARHGAP35 expression had a better prognosis (Fig. 4I). Finally, we further evaluated the prognostic value and found that the score for the three biomarkers combined was significantly associated with OS (Fig. 4J), and these findings suggest that low expression of ARHGAP35 in combination with high expression of FTO and ALKBH5 may serve as a vital clinical biomarker of poor prognosis in NPC patients.

These results suggested that ARHGAP35, as a tumor suppressor, was upregulated after coknockdown of FTO and ALKBH5, and this phenotype predicted a better prognosis for NPC patients.

### m^6^A maintains the mRNA stability and translation of ARHGAP35 in NPC cells

**Fig. 5:**
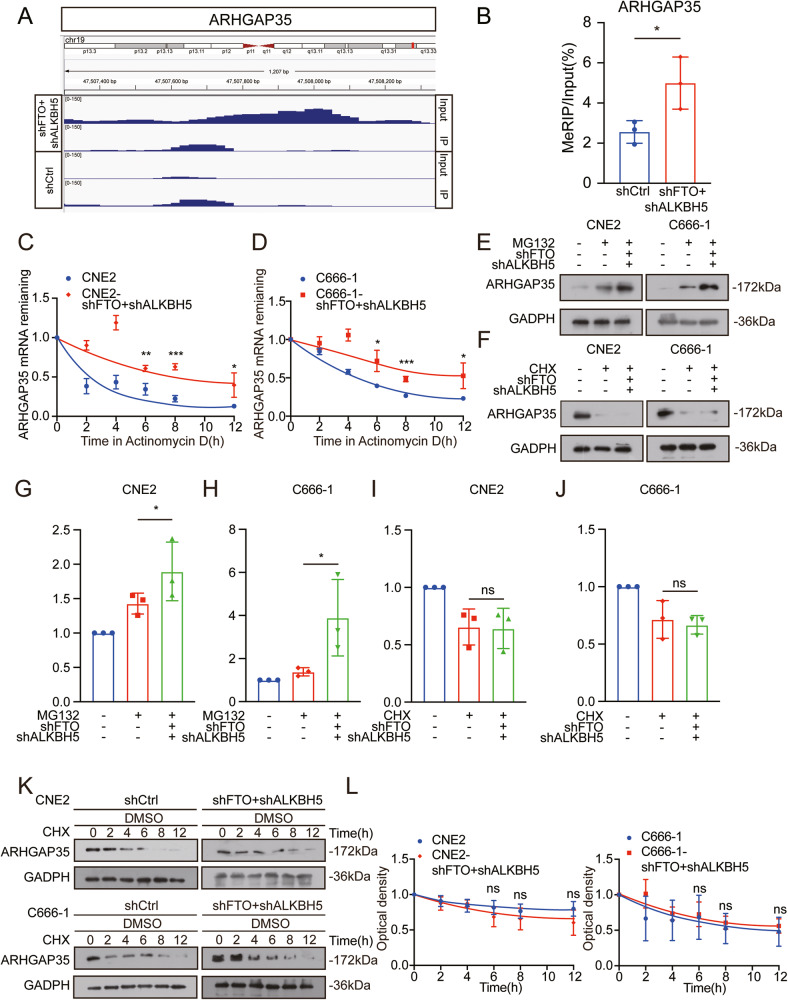
It promotes the stability of ARHGAP35 mRNA and translation by jointly downregulating FTO and ALKBH5 related m^6^A modification. **A** The m^6^A peak showed the relative abundance of the m^6^A sites on ARHGAP35 mRNA in shFTO+shALKBH5 CNE2 cells. **B** Detection of m^6^A enrichment in endogenous ARHGAP35 mRNA in NPC cells. Using MeRIP method, quantitative RT-PCR was used to detect the ARHGAP35 mRNA level in the same number of NPC cells with and without FTO, ALKBH5 combined knockdown. *p < 0.05, student’s test. **C**, **D** The stability of ARHGAP35 mRNA in CNE2 and C666-1 cells was determined by RT-qPCR after treatment with transcription inhibitor: actinomycin D (ActD). GADPH is used as internal parameter. The data were expressed as the average ± SD value of the three biological repeats. *p < 0.05, **p < 0.01, and ***p < 0.001, student’s test. **E**–**J** NPC cells were pre-transfected with vector control or FTO construct for 24 h and then further treated with CHX (10 μg/ml) or MG-132 (5 μM) for 6 h, the expression of ARHGAP35 was detected by western blot analysis (up) and quantitatively analyzed (down). **K**, **L** Western Blot assay was used to detect the protein expression of ARHGAP35 in CNE2 and C666-1 cells treated with cycloheximide (CHX) for the indicated time periods (left) and quantitatively analyzed (right).

We further investigated the potential mechanism by which m^6^A modification regulates the expression of ARHGAP35. From the m^6^A-seq data of shFTO+shALKBH5 NPC cells, we found a statistically significant and unique m^6^A peak in ARHGAP35 mRNA, which was located on its CDS (Fig. 5A). This unique peak is specifically synergistically demethylated by FTO and ALKBH5, indicating that its functional correlation is regulated by m^6^A. At the same time, to directly confirm the existence of m^6^A modification in the CDS on ARHGAP35 mRNA, we conducted an m^6^A-RIP experiment in NPC cells. Compared with shCtrl cells, the level of m^6^A in ARHGAP35 mRNA was significantly enriched after coordinated knockdown of FTO and ALKBH5 (Fig. 5B), which supports the view that m^6^A-modified ARHGAP35 mRNA is synergistically regulated by FTO and ALKBH5.

We treated shFTO+shALKBH5 and shCtrl NPC cells with Act-D to block transcription. The mRNA stability of ARHGAP35 in shFTO+shALKBH5 NPC cells (Fig. 5C, D) was significantly greater than that in their corresponding shCtrl cells. This result indicated that m^6^A modification may reduce the degradation of ARHGAP35 mRNA in NPC cells.

Moreover, we further explored whether m^6^A can regulate the expression of ARHGAP35 but not its mRNA stability. Both NPC cell lines transfected with shFTO+shALKBH5 or shCtrl were further treated with MG132 to restrain proteasome activity or with cycloheximide (CHX) to inhibit protein translation. This result showed that in the presence of MG132 but not CHX, FTO and ALKBH5 coinduced the protein expression of ARHGAP35 in NPC cells (Fig. 5E–J), suggesting that m^6^A modification might affect the protein translation rather than the stability or posttranslational modification of ARHGAP35. This was verified by the result that the half-life of the ARHGAP35 protein was not significantly different between shFTO+shALKBH5 and shCtrl NPC cells (Fig. 5K–L).

These results indicate that m^6^A modification maintains the mRNA stability and translation of ARHGAP35 in NPC cells.

### Overexpression of ARHGAP35 effectively inhibits the malignant progression of NPC in vitro and in vivo

To explore the role of ARHGAP35 in the malignant progression of NPC, we overexpressed ARHGAP35 in CNE2 and C666-1 cells (Figure S3A, B) and then found that NPC cells overexpressing ARHGAP35 showed significant inhibitory effects on proliferation and migration. The above experimental conclusions showed that cells overexpressing ARHGAP35 showed reduced EdU incorporation and colony formation ability (Figure S3C–F), and a similar trend was confirmed by transwell and wound healing tests (Figure S3G-J). Through flow cytometry analysis, we found that overexpression of ARHGAP35 can promote the occurrence of apoptosis (Figure S3K, L). These results showed that overexpression of ARHGAP35 inhibited the proliferation and migration of NPC cells.

**Fig. 6:**
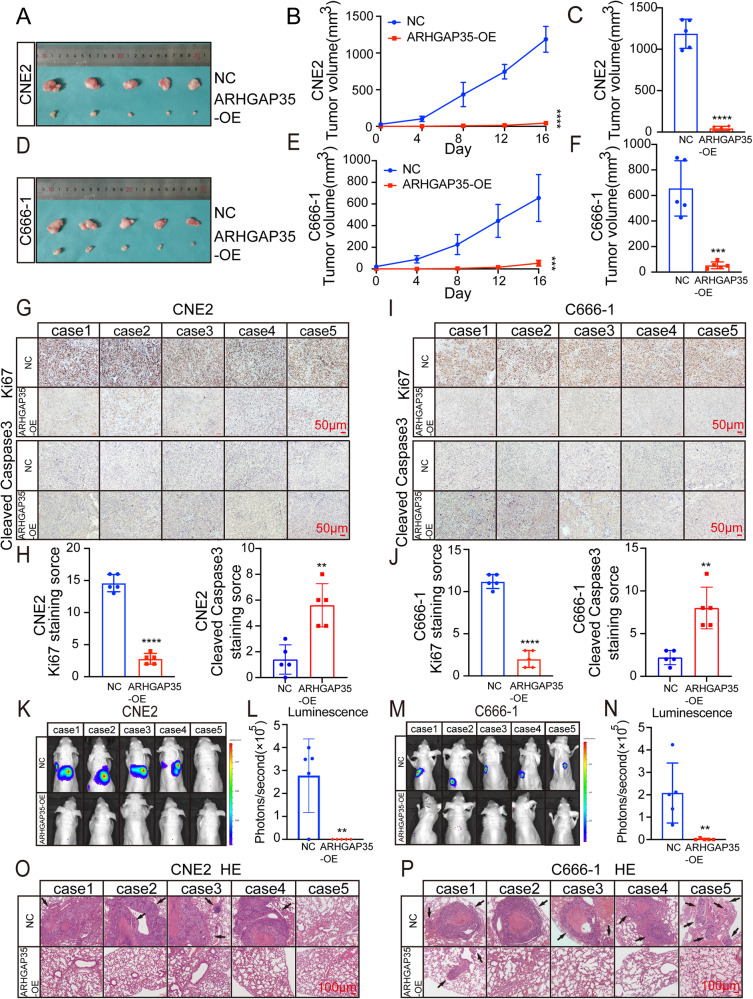
Overexpression of ARHGAP35 inhibits the growth and metastasis of NPC in vivo. **A**, **D** The corresponding images of xenogeneic tumor grafts. **B**, **E** After being injected with NPC cells, the tumor volume of the two groups was compared. (***p < 0.001, ****p < 0.0001, one-way ANOVA). **C**, **F** The volume of xenograft removed. (***p < 0.001, ****p < 0.0001, one-way ANOVA). **G**, **I** Representative images of Ki67 and Cleaned Caspase3 IHC in xenograft tissues of NPC in two groups. **H**, **J** IHC staining score of Ki67 and Cleaned Caspase3 expression. (****p < 0.0001, one-way ANOVA). **K–N** The lung metastasis in BALB/c mice have been visualized and quantitative analyzed. (**p < 0.01, one-way ANOVA). **O**, **P** HE staining image of metastatic lung tissue. Scale: 100 μm.

Based on the effects of ARHGAP35 on the proliferation and migration of CNE2 and C666-1 cells observed in vitro, we further conducted and analyzed in vivo experiments with 4-week-old nude mice. We subcutaneously injected CNE2 and C666-1 NPC cell lines and control cells overexpressing ARHGAP35 into nude mice. The tumor volume of nude mice in the shCtrl group was measured on the 4th, 8th, 12th and 16th days after the formation of subcutaneous tumors (Fig. 6A–F). The xenografts were removed, and the volume was measured after 16 days (Fig. 6C, F). We found that the volume of xenografts overexpressing ARHGAP35 was much smaller than that of the xenografts in the control group (Fig. 6A–F). The staining of Ki67 and Cleaned Caspase3 by IHC also reflects that overexpression of ARHGAP35 inhibits the growth of NPC by promoting apoptosis (Fig. 6G–J). Similarly, in vivo imaging experiments and subsequent HE staining of lung tissues in nude mice showed that overexpression of ARHGAP35 inhibited the lung metastasis of NPC cells in vivo (Fig. 6K–P).

In conclusion, these experiments confirmed that ARHGAP35 is a tumor suppressor and inhibits malignant progression in NPC.

### The FTO/ALKBH5-ARHGAP35 signaling axis promotes the proliferation and migration of NPC

To further confirm whether ARHGAP35 is downstream of both FTO and ALKBH5, we knocked down both FTO and ARHGAP35 and blocked the expression of ARHGAP35 in NPC cells (Figure S4A–D). We wanted to determine whether inhibition of ARHGAP35 can reverse FTO and ALKBH5 coknockdown in tumors in vivo and in vitro. Through EdU and cell colony experiments, we found that downregulation of ARHGAP35 promoted the growth of NPC cells with FTO and ALKBH5 knockdown in vitro (Figure S4E–H). Knockout of ARHGAP35 in transwell and wound healing experiments also reversed the ability of shFTO+shALKBH5 to inhibit tumor cell migration (Figure S4I–L). These results showed that knockout of ARHGAP35 reversed the ability of shFTO+shALKBH5 to promote apoptosis by flow cytometry (Figure S4M–N).

**Fig. 7:**
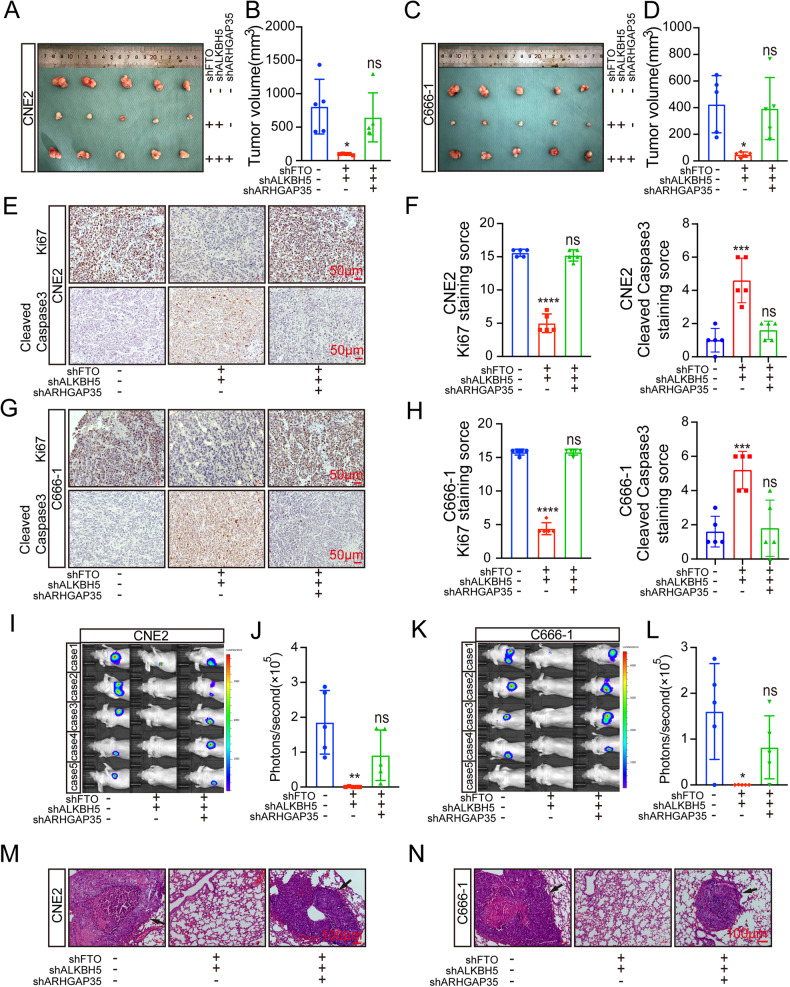
In vivo experiment verifies that FTO/ALKBH5-ARHGAP35 signal axis promotes the malignant behavior of NPC. **A**, **C** Images of subcutaneous xenograft tumor in BALB/c mice. **B**, **D** The volume of xenografts removed by surgery. (*p < 0.0001, one-way ANOVA). **E**–**H** representative image and staining score of Ki67 and Cleaned Caspase3 after IHC staining of xenograft. (****p < 0.0001, one-way ANOVA). **I**–**L** Visualization and analysis of mouse lung metastasis model. (*p < 0.05, **p < 0.01, one-way ANOVA) (**M**, **N**) HE staining image of metastatic tumor of lung tissue. Scale: 100 μm.

**Fig. 8:**
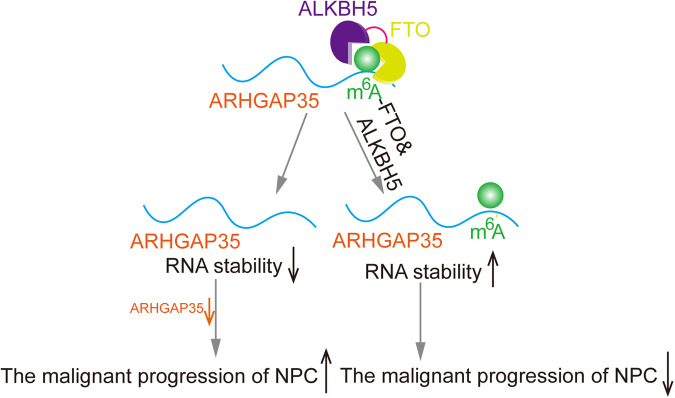
Schematic diagram of the effect of FTO and ALKBH5 on promoting the malignant phenotype of NPC by negatively regulating the m^6^A modification level.

In addition, through subcutaneous tumorigenesis and IHC staining assays in nude mice, we found that downregulation of ARHGAP35 could reverse the inhibition of NPC tumor growth by promoting apoptosis in nude mice after combined knockout of FTO and ALKBH5 (Fig. 7A–H). Similarly, in vivo imaging experiments and HE lung tissue staining experiments revealed that shFTO+shALKBH5 tumor metastasis of NPC in nude mice could be reversed by downregulation of ARHGAP35 (Fig. 7I–N). Combining these conclusions with the previously confirmed conclusion supports that the m^6^A level in ARHGAP35 is regulated by shFTO+shALKBH5. We can conclude that in NPC, ARHGAP35 can act codownstream of both FTO and ALKBH5 and promote the proliferation and migration of NPC cells through the FTO/ALKBH5-ARHGAP35 axis (Fig. 8).

## Conclusion

Low expression of the m^6^A demethylases FTO and ALKBH5 inhibits the malignant process of NPC by regulating m^6^A modification. The inhibitory effect of FTO/ALKBH5 knockdown alone on the NPC malignant phenotype was not as significant as that of the combined knockdown of FTO and ALKBH5, and even their inhibitory function could be reversed. ARHGAP35 is the downstream target of the synergistic regulation of shFTO and shALKBH5 and acts as a tumor suppressive factor to inhibit the malignant biological behavior of NPC. These findings provide new insights into the molecular regulatory mechanism of NPC progression and new strategies for the clinical treatment of NPC.

## Discussion

Some progress has been made in research on m^6^A modification in NPC. m^6^A modification plays a key role in the growth and metastasis of NPC tumors by regulating the immune microenvironment of tumor cells [29]. Therefore, m^6^A modification is considered an important RNA modification that runs through the whole process from transcription to translation regulation [31]. m^6^A modification can regulate the expression of downstream targets by maintaining the stability of coding and noncoding RNAs in NPC cells [30, 32]. The m^6^A demethylases FTO and ALKBH5 can promote the progression of HNSCC tumors [12, 13].

However, FTO and ALKBH5 are rarely studied in NPC. A recent breakthrough study confirmed that the expression of FTO is significantly increased in radiotherapy-resistant NPC cells and tissues and that FTO triggers radiotherapy resistance in NPC by reducing the m^6^A modification of OTUB1 [19, 33]. Unfortunately, the effect of FTO on the aggressive behavior of NPC has not been reported, and ALKBH5 has not been studied in detail. Moreover, the role of m^6^A in NPC is also unclear. We believe that it is necessary to explore how FTO and ALKBH5 affect the growth and metastasis of NPC by regulating m^6^A modification, which will provide researchers with deeper insights into the role of m^6^A in NPC and play a positive role in the prognosis of NPC patients.

Based on clinical samples and histological analysis, we confirmed that m^6^A expression was low in NPC patients. The expression of FTO and ALKBH5 was significantly increased in patients with advanced TNM stage, and the levels of FTO and ALKBH5 were significantly correlated with the prognosis of patients. Importantly, a positive correlation was found between FTO and ALKBH5 levels in NPC tissue samples. Subsequently, in the xenotransplantation model of nude mice, it was found that knockdown of FTO or ALKBH5 expression could inhibit the growth and metastasis of NPC, while knockdown of a single demethylase alone had a short-acting effect on NPC, and their inhibitory effects decreased and even disappeared with time. Moreover, the co-knockdown of both FTO and ALKBH5 exerted a stable inhibitory effect.

With the development and application of high-throughput sequencing technology, the study of m^6^A modification in tumors has become more in depth [34, 35]. Using sequencing technology and bioinformatics analysis, we found that the downstream target gene in NPC, ARHGAP35, is jointly regulated by FTO and ALKBH5. Data from the human protein map database indicate that ARHGAP35 is involved in transcription and transcriptional regulation. ARHGAP35, as a tumor suppressor, induces CDH1 expression and cooperates with E-cadherin to activate LATS kinase and inhibit tumor cell growth [36]. Its role in NPC has not been reported. We found a unique m^6^A peak on the CDS of ARHGAP35 through m^6^A-seq data and confirmed through MeRIP that the m^6^A level of ARHGAP35 in shFTO+shALKBH5 NPC cells was significantly increased. Then, through RNA and protein stability experiments, it was found that FTO combined with ALKBH5 could effectively affect the attenuation of ARHGAP35 mRNA and protein. Therefore, we determined that FTO and ALKBH5, which are joint upstream of and regulated by ARHGAP35, can inhibit the malignant progression of NPC by inhibiting cell growth and metastasis. This has deepened our understanding of the inhibition of NPC by ARHGAP35.

At present, the efficacy of treatments for metastasis or recurrence of NPC is still unclear. Our data provide important progress in understanding the importance and mechanism of m^6^A modification in the transcriptional regulation and malignant progression of NPC. Therefore, our findings provide a new prognostic indicator for NPC patients and a new idea for gene targeted therapy.

## Materials and methods

### Tissue samples from NPC patients

With the approval of the Ethics Committee and the informed consent from all the subjects, tumor tissues of NPC patients confirmed by pathology were collected in the Affiliated Hospital of Nantong University. Tumor tissue samples were stored at −80 °C before being used. The expression of m^6^A modification in NPC tissues, which did not receive any treatment before biopsy. were detected by dot blot and colorimetry.

### Cell lines and cell culture

Human NPC cell lines (CNE2, C666-1) were cultured at the Otoh Research Institute in the Department of Otorhinolaryngology Head and Neck Surgery, Affiliated Hospital of Nantong University (Jiangsu, China). They were cultured in RPMI 1640 (Biological Industries Israel Beit-Haemek, 01–100-1ACS) containing 10% FBS (Biological Industries Israel Beit-Haemek, 04–001-1ACS). The cells were grown at 37 °C and 5% CO_2_. Before the experiment, the cells were confirmed to be free of mycoplasma contamination.

### Western blotting

Cells were collected and lysed in protein lysis buffer, and the concentration was measured using a BCA kit (Thermo Fisher Science, 23327). Protein samples (40 μg) were subjected to electrophoresis and transferred to nylon membranes, which were sealed with blocking buffer. Finally, the cells were incubated with the primary antibody at 4 °C for 6 h. The antibodies used were anti-FTO (cat: 27226-1-AP, Proteintech, China), anti-ALKBH5 (cat: 16837-1-AP, Proteintech, China), and anti-ARHGAP35 (cat: 26789-1-AP, Proteintech, China).

### qRT‒PCR

Total RNA was extracted on ice with TRIzol reagent (cat: B511311, Sangon Biotech, China), and total RNA samples were resuspended in water without RNA. A reverse transcriptase kit was used for reverse transcription (Thermo Fisher Science, K1622), and qRT‒PCR was performed using SYBR Green PCR Master Mix (Roche, 04913914001). The sequences of all indicator primers are as follows:

FTO-forward: GTT CAC AAC CTC GGT TTA GTT C

FTO-reverse: CAT CAT CAT TGT CCA CAT CGT C

ALKBH5-forward: CGG ACC TGC GTG AGA AG

ALKBH5-reverse: TCC TGA TAC TTG CGC TTG G

ARHGAP35-forward: GAA ATT GAC GGA AGG TTC ACA A

ARHGAP35-reverse: CGG CAG CAT TAT CGT ACA TAT G.

### Lentiviral vector transfection

Three short hairpin RNAs (shRNAs) targeting human FTO, ALKBH5 and ARHGAP35 and one overexpression lentivirus targeting human ARHGAP35 and its negative control were constructed using plasmids and lentiviral vectors and were generated by GeneChem (Shanghai, China). The three short hairpin RNA sequences are shown in the following table:

shFTO: ctAGAAGGAGCACAAGTCTCA

shALKBH5: ccACCCAGCTATGCTTCAGAT

shARHGAP35: cgGTTGGTTCATGGGTACATT

We transfected cells with LV-FTO/ALKBH5/ARHGAP35-GFP (GV218; GeneChem) for 24 h.

### m^6^A quantization

The overall level of m^6^A in mRNA was detected by using the m^6^A RNA Methylation Assay kit (colorimetry) (Abcam, ab185912). Each sample was analyzed with 200 ng poly-A purified RNA.

### MeRIP, RNA sequencing and MeRIP-qPCR

The MeRIP detection method has been widely reported [37]. MeRIP sequencing and RNA sequencing were performed by Cloudseq Biotech, Inc. (Shanghai, China).

Briefly, total RNA was extracted from four groups of CNE2 cells, shCtrl, shFTO, shALKBH5, and shFTO+shALKBH5 cells, using TRIzol reagent. Next, the total RNA was digested into 100 nt fragments, purified into complete poly-A RNA, denatured at 70 °C for 10 min, and then processed according to the MagnaRIP™ kit (Millipore) according to the instructions. The RNA immunoprecipitated by the anti-m^6^A antibody (Synaptic Systems, 202003) was reverse transcribed, and then the m^6^A expression in ARHGAP35 was measured by qRT‒PCR using the primer of ARHGAP35.

### Dot blot

Total RNA or poly (A)+mRNA was isolated from tissue samples (n = 3) of normal people and NPC patients. Then, the sample was divided into two parts, both quantitative to 200 ng, and placed into an Amersham Hybond-N+ membrane (GE Healthcare, USA). The membranes were cross-linked by ultraviolet light under a vacuum environment for 5 min and washed with PBST. After that, one of them was dyed with 0.01% methyl blue (China Sangong Biotechnology Co., Ltd. and then scanned to indicate the total content of input RNA. After being blocked with 5% skim milk, methyl blue stain was applied, and then the membrane was incubated at 4 °C with a specific m^6^A antibody (1:1000, mmol) for 12 h. The samples were incubated with HRP-bound anti-mouse immunoglobulin G (IgG) for 1 h, and then an imaging system was used for visual analysis (Bio-Rad, USA).

### Stability assay

We inoculated 1 × 10^7^ cells/2 mL complete culture medium into a 6-well plate (NEST, 703001), and 10 μM cycloheximide (CHX, Monmouth Junction, NJ, USA) was added for a specified time (0, 2, 4, 6, 8, and 12 h) to block protein synthesis; 20 μM MG132 was also given to inhibit the proteasome for 6 h before harvest to block protein degradation. Then, the protein was extracted, and the protein expression of ARHGAP35 was detected by western blotting to detect the stability of protein translation. GAPDH was used as a control.

Similarly, 5 g/mL actinomycin D (ActD, Monmouth Junction, NJ, USA) was added to the above equivalent cells to inhibit mRNA transcription. After the same treatment for a specified time (0, 2, 4, 6, 8 and 12 h), the RNA at the corresponding time point was collected, and then the RNA expression of ARNGAP35 was measured by qRT‒PCR to detect the RNA stability. Nondegraded 18 S rRNA was used as a control.

### Cell proliferation and migration test

To analyze cell proliferation by using the cell colony formation experiment, we inoculated 100 cells/2 mL complete culture medium into a 6-well plate. After the cells were incubated at 37 °C in a CO_2_ concentration incubator for 2 weeks, they were fixed with paraformaldehyde and stained with crystal violet dye, and then images were collected.

For the EdU cell proliferation experiment, 6 × 10^3^ cells/200 μL complete medium was inoculated into 96-well plates (NEST, 701001), and the cells cultured for 24 h were labeled with EdU reagent. Images were captured under an inverted microscope.

For the transwell experiment, 6 × 10^4^ cells/200 μL RPMI 1640 medium was inoculated into the chamber of a 24-well plate (NEST, 702001), and 500 μL complete medium was added to the lower well. After 18 h, remove the chamber, erase the internal cells, fix the sample and then capture the photos.

In the wound healing experiment, the cells were cultured in 6-well plates until they were nearly confluent of the bottom surface, and the complete medium was replaced with serum free RPMI 1640 medium. Then, a “+” was drawn with the tip of a 200 mL sterile pipette, and an image was taken. After 48 h of starvation, another image of the same region was taken.

### Flow cytometry analysis

Apoptosis of NPCs were detected using the Annexin V-keyFlour647 Apoptosis/PI Detection Kit (KeyGEN Bio-TECH Co., Ltd.). Briefly, 2 × 10^5^/ Test of NPCs were resuspended in 500 μL binding buffer, and then labeled with Annexin V-kFlour647 (5 μL) and propidium iodide (PI) (5 μL) for 10 min in the dark at 25 °C. After 45 min, the red (Annexin V-kFlour647) and red (PI) fluorescence were examined by flow cytometry (MilliporeSigma Co., Ltd., Burlington, MA, USA).

### In vivo analysis using BALB/c nude mouse animal models

To effectively assess the significant differences in the growth and metastasis of cells in each group in vivo, we subcutaneously injected 1 × 10^7^ cells (FTO/ALKBH5/ARHGAP35 knockdown CNE2 cells or ARHGAP35-expressing C666-1 cells) in 200 μL RPMI 1640. Every 4 days, Vernier calipers were used to record the size of the tumor under the skin of the 4-week-old nude mice. Then, the tumor growing under the skin was removed, fixed with 10% formalin and cut into paraffin sections for subsequent IHC detection.

With similar cell groups, we injected 1 × 10^6^ luciferase-labeled CNE2 and C666-1 cells/200 μL RPMI 1640 medium into the tail vein of 7-week-old nude mice (n = 5 for each group). IVIS Lumina Series III (Calper Life Sciences, Mountain View, CA, USA) was used to measure the photon flux of the whole body of mice every week to confirm the success of cell injection. During the course of 1 month, we used bioluminescence imaging (BLI) to analyze the lung metastasis of each mouse. Finally, after the nude mice were killed, the lung tissue of each mouse was dissected, soaked in 10% formalin, made into paraffin sections of lung tissue, and sent to the Pathology Department of Nantong University Affiliated Hospital for HE staining.

In one animal experiment, we selected nude mice of the same sex (male), same age, and similar weight to minimize the impact of sample differences on experimental results. The group and type of cells are unknown to the experimenters when they inject them.

### Immunohistochemistry (IHC)

As previously mentioned, FTO, ALKBH5, ARHGAP35 and Ki67 expression were analyzed by immunohistochemistry. The staining intensity score was divided into 4 grades: 1 (negative), 2 (weak positive), 3 (medium positive), and 4 (strong positive). The staining area score was divided into 4 grades: 1 (0–25%), 2 (26–50%), 3 (51–75%), and 4 (>75%). The product of the two scores was the final staining score (low expression: 0–8 points, high expression: 9–16 points). In addition to the above western blotting antibodies, anti-Ki67 antibody (cat: 27309-1-AP, Proteintech, China) and anti-cleaved Caspase 3 (cat: 25128-AP, Proteintech, China) was used in these studies.

### Statistical analysis

The statistical analysis software used in this study was GraphPad Prism 7 and SPSS 20.0. The results of at least three independent experiments are the average ± standard error of the mean (SEM) or standard deviation (SD). The correlation analysis used in this study was Spearman’s rank correlation coefficient. A Kaplan‒Meier survival curve was used for survival analysis with the log-rank test. The statistical significance was determined by one-way ANOVA and two-tailed Student’s *t* test. *p* < 0.05 was considered to indicate statistical significance.

### Supplementary information

Supply Figure legends

Supply Figure 1

Supply Figure 2

Supply Figure 3

Supply Figure 4

Original Data File

Supply Table

## Data Availability

The datasets presented in this study can be provided to editors if necessary.
