# Supplementary material for: The m6A demethylases FTO and ALKBH5 aggravate the malignant progression of nasopharyngeal carcinoma by coregulating ARHGAP35
Source: Cell Death Discov. 2024 Jan 23;10:43. doi: 10.1038/s41420-024-01810-0 (PMC10806234; doi:10.1038/s41420-024-01810-0)
Supplement: Supplementary file 1 — Supply Figure legends [file 41420_2024_1810_MOESM1_ESM.doc]

**Supply Figure**

**Supply Fig1** **NPC cell lines (CNE2, C666-1) treated with lentivirus: shFTO and shALKBH5.**

A, C, D: The protein expression of FTO and ALKBH5 in CNE2 and C666-1 cells after FTO and ALKBH5 knockdown was verified individually or jointly by Western blot. B, E-H: RT-qPCR was used to verify RNA levels of FTO and ALKBH5 in CNE2 and C666-1 cells after knockdown of FTO and ALKBH5 individually or jointly. I: The level of apoptosis in CNE2 and C666-1 cells after knockdown of FTO and ALKBH5 individually or jointly was analyzed by flow cytometry. **p*<0.05, ***p*<0.01, *****p*<0.0001, one-way ANOVA. The data represents the average ± SEM.

**Supply Fig2** **Low expression of FTO and ALKBH5 synergistically inhibit the proliferation and migration of NPC in vitro.**

A, C: The CNE2 and C666-1 cells of shCtrl, shFTO, shALKBH5, shFTO+shALKBH5 were cultured to the 4th day, and EdU was detected 10 days later. Cells that had not been transfected with shFTO and shALKBH5 on day 0 were used as control. B, D: Randomly select (A, C) EdU-positive NPC cells in the microscopic field. n=3. E, G: Like EdU, transwell detected shCtrl, shFTO, shALKBH5, shFTO+shALKBH5 CNE2 and C666-1 cells on the 4th and 10th day. G. H: The number of cells invading the cell membrane was calculated in three fields of vision in each group, and there were three independent repeats. **p*<0.05, ***p*<0.01, ****p*<0.001, *****p*<0.0001, one-way ANOVA. The data represents the average ± SEM.

**Supply Fig3 ARHGAP35 inhibits proliferation and migration of NPC cells.**

A, B: Western Blot and RT-qPCR validated the efficiency of ARHGAP35 in transfection of Lv-ARHGAP35 in NPC cells. *****p*<0.0001. C, D: Left: Colony formation test of NPC cells overexpressing ARHGAP35. Right: The number of NPC cell colonies. N=3. ***p*<0.01, ****p*<0.001, student’s test. E, F: Above: The proliferation of NPC cells was evaluated when ARHGAP35 was overexpressed by adding EdU (5-Ethynyl-2’- deoxyridine) for 2 hours. Scale, 100μm. Below: NPC cells with EdU positive were randomly selected and quantified under microscope. n=3, **p*<0.5, ****p*<0.001. student’s test. G, H: Above: transwell method was used to detect cell migration. The scale represents 100μm. Below: Statistical comparison of the number of cells detected by the two groups. ****p*<0.001. I, J: Left: The cell migration was measured by wound closure test. The scale represents 200μm. Right: Statistical comparison of the percentage of wound width between the two groups. ***p*<0.01, ****p*<0.001, student’s test. K, L: Flow cytometry analyse was used to show the level of apoptosis in CNE2 and C666-1 cells after overexpressing ARHGAP35. ****p*<0.001, *****p*<0.0001.

**Supply Fig4 FTO/ALKBH5-ARHGAP35 signal axis promotes the proliferation and migration of NPC cells in vitro.**

A-D: The expression of ARHGAP35 was verified in NPC cells after silencing ARHGAP35 by Western Blot and RT-qPCR individually or jointly. **p*<0.05, ***p*<0.01. E: Colony formation test of NPC cells down-regulating FTO, ALKBH5 or ARHGAP35. F: The number of NPC cell colonies. N=3, ***p*<0.01, ****p*<0.001, *****p*<0.0001, one-way ANOVA. G: The proliferation of NPC cells in down-regulation of FTO, ALKBH5 or ARHGAP35 was evaluated by EdU incorporation for 2 hours. Scale: 100μm. H: EdU-positive NPC cells were randomly selected and quantified under microscope. N=3, ***p*<0.01, ****p*<0.001, one-way ANOVA. I: The wound closure test measured the migration of NPC cells when FTO, ALKBH5 or ARHGAP35 were down-regulated. Scale: 200μm. J: Statistical comparison of the percentage of wound width. *****p*<0.0001, one-way ANOVA. K: Cell migration was detected by transwell method. Scale: 100μm. L: Statistical comparison of cell number by pore detection ***p*<0.01, ****p*<0.001, *****p*<0.0001. one-way ANOVA. M: Analysis of apoptosis-related images by flow cytometry. N: Statistical comparison of the level of apoptosis. **p*<0.05, *****p*<0.0001. one-way ANOVA.

**Supply Fig5 Uncropped blots for three independent experiments presented in the figures.**

A: Immunoblots of cropped blots as shown in **Figure S1A, C and D; Fig. 4A.** B: Immunoblots of cropped blots as shown in **Fig. 5E, F, K**. C: Immunoblots of cropped blots as shown in **Figure S3A.** D: Immunoblots of cropped blots as shown in **Figure S4A**. E: Immunoblots of cropped blots as shown in **Figure S4C**.
