# Supplementary figures and images for: The m6A demethylases FTO and ALKBH5 aggravate the malignant progression of nasopharyngeal carcinoma by coregulating ARHGAP35
Source: Cell Death Discov. 2024 Jan 23;10:43. doi: 10.1038/s41420-024-01810-0 (PMC10806234; doi:10.1038/s41420-024-01810-0)

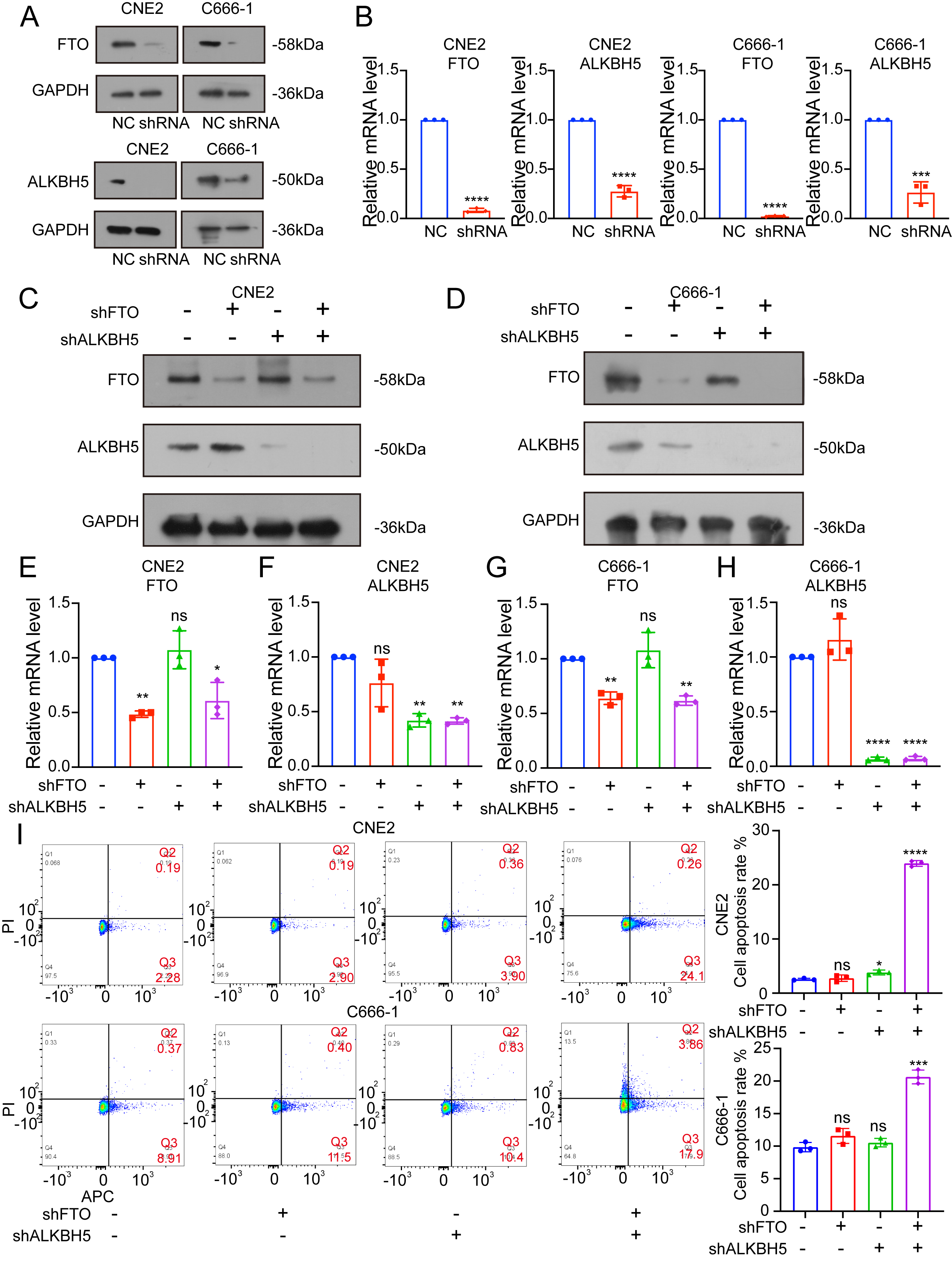

Supplement: Supplementary file 2 — Supply Figure 1 [file 41420_2024_1810_MOESM2_ESM.tif]

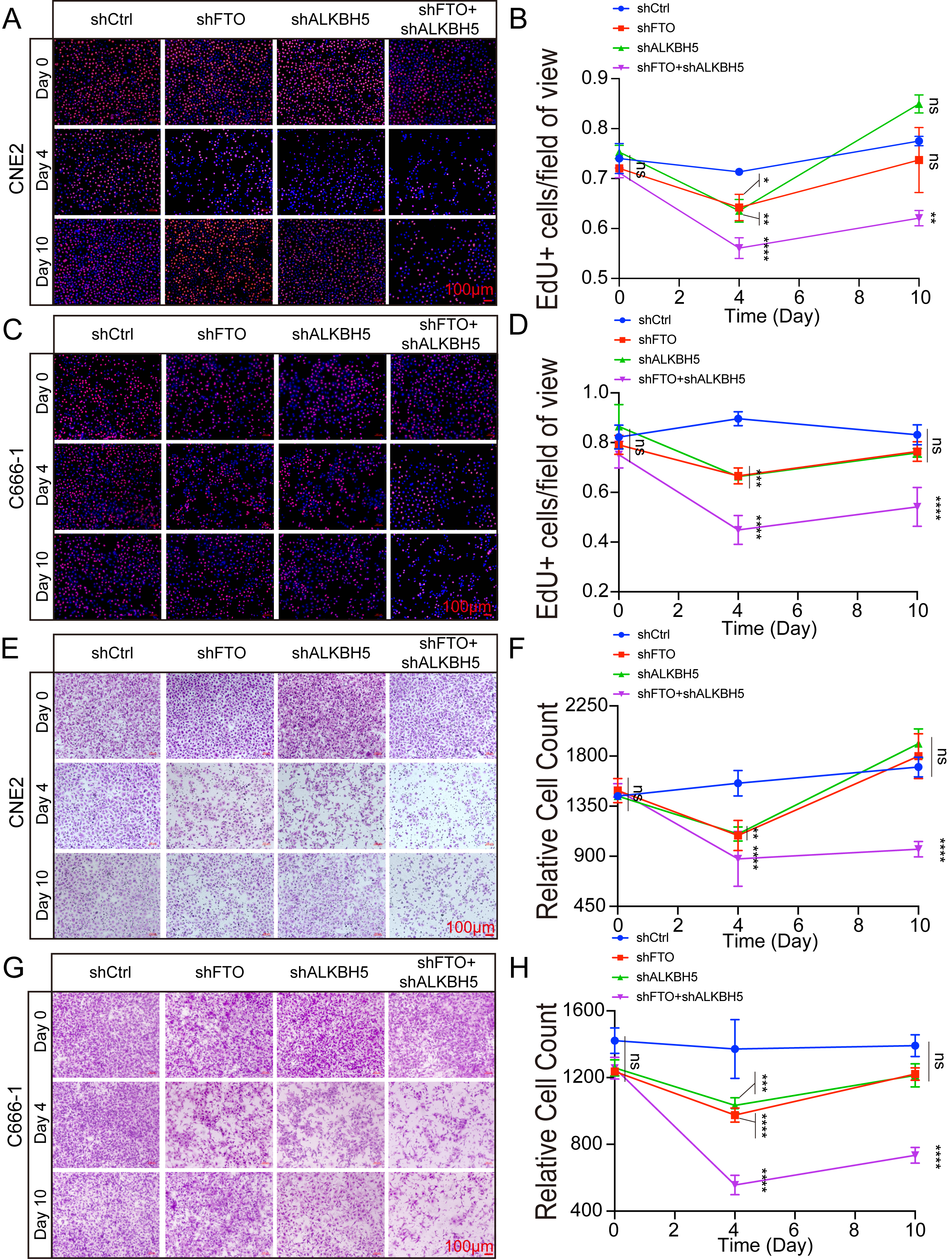

Supplement: Supplementary file 3 — Supply Figure 2 [file 41420_2024_1810_MOESM3_ESM.tif]

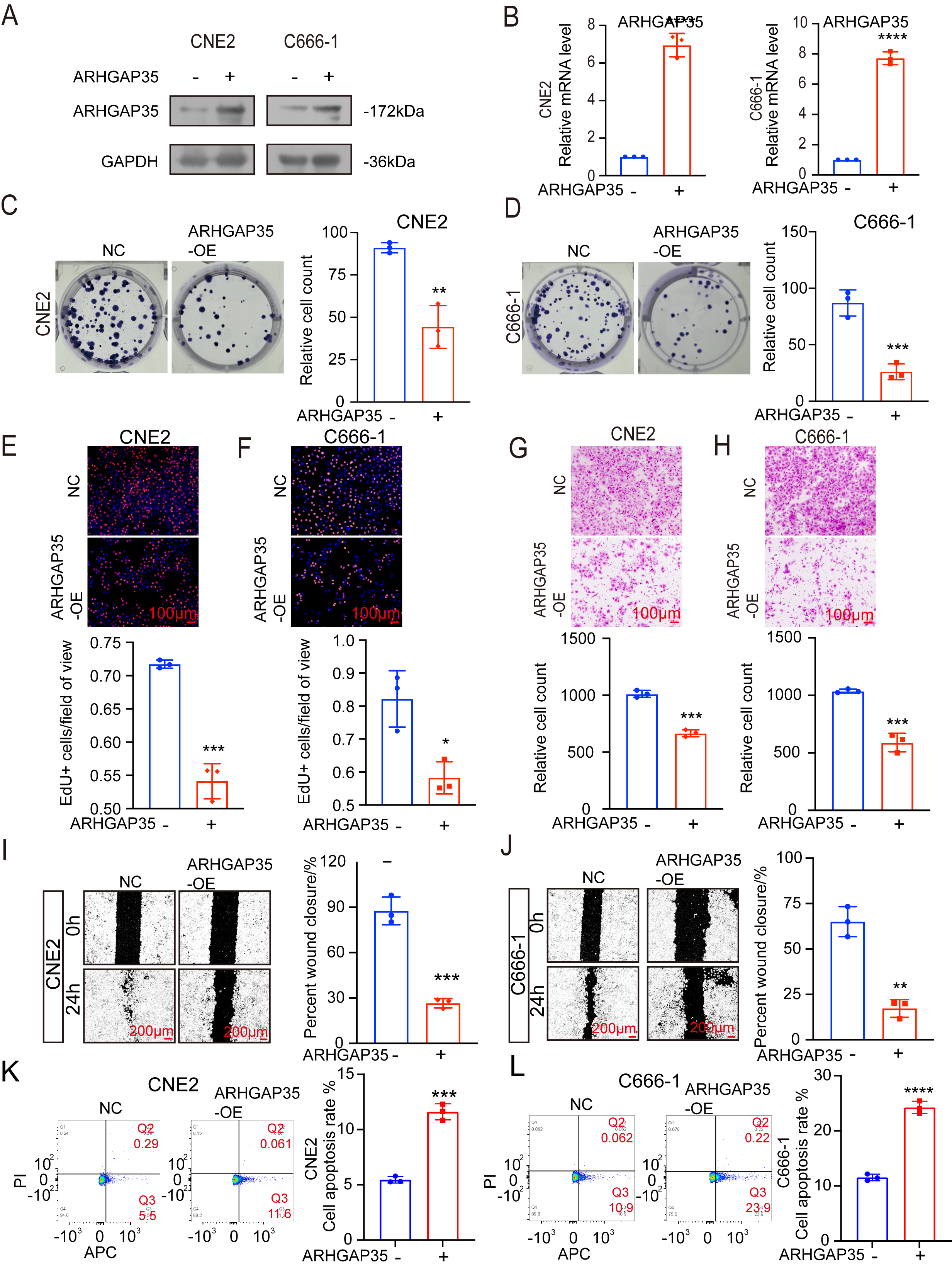

Supplement: Supplementary file 4 — Supply Figure 3 [file 41420_2024_1810_MOESM4_ESM.tif]

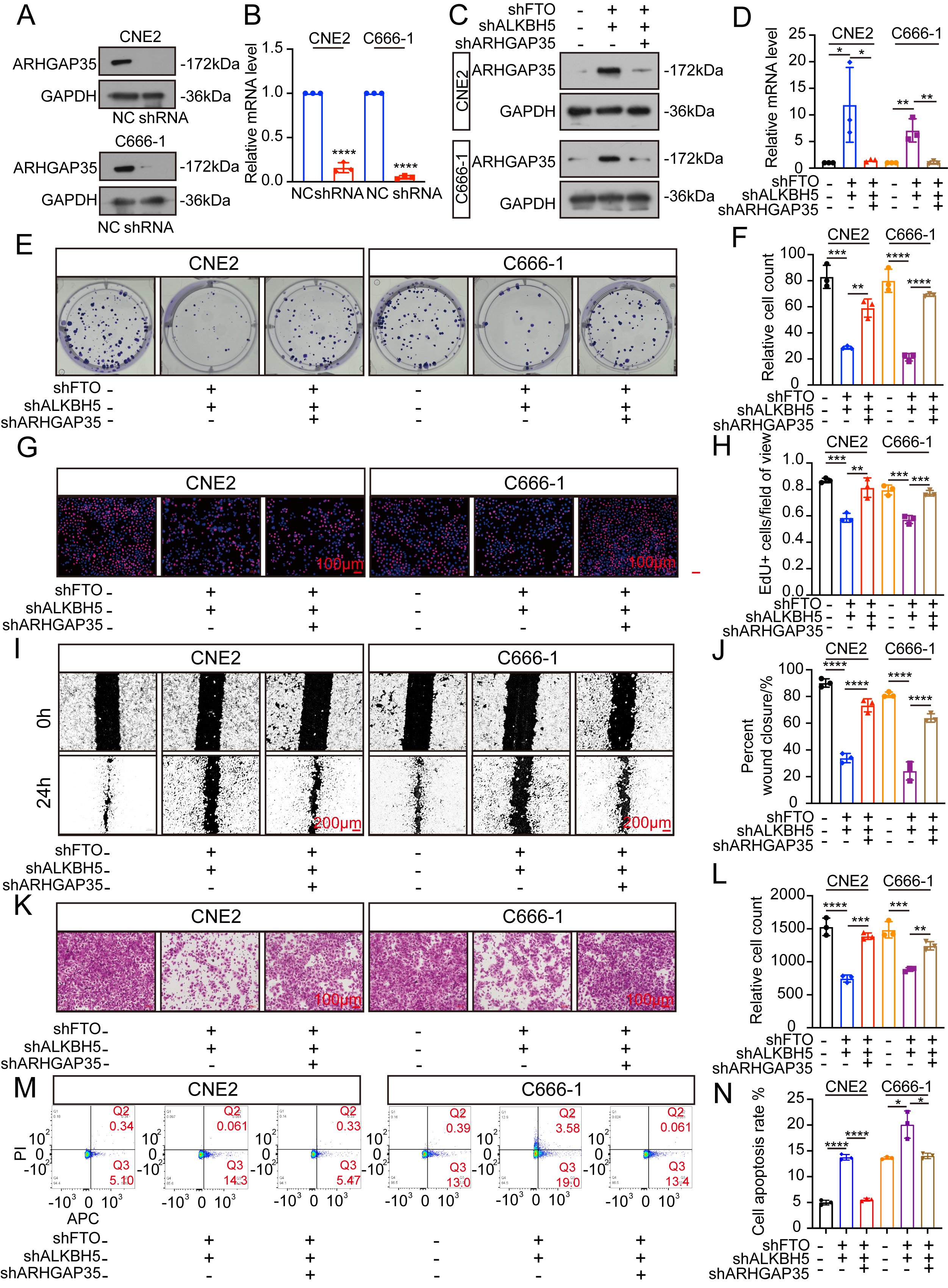

Supplement: Supplementary file 5 — Supply Figure 4 [file 41420_2024_1810_MOESM5_ESM.tif]

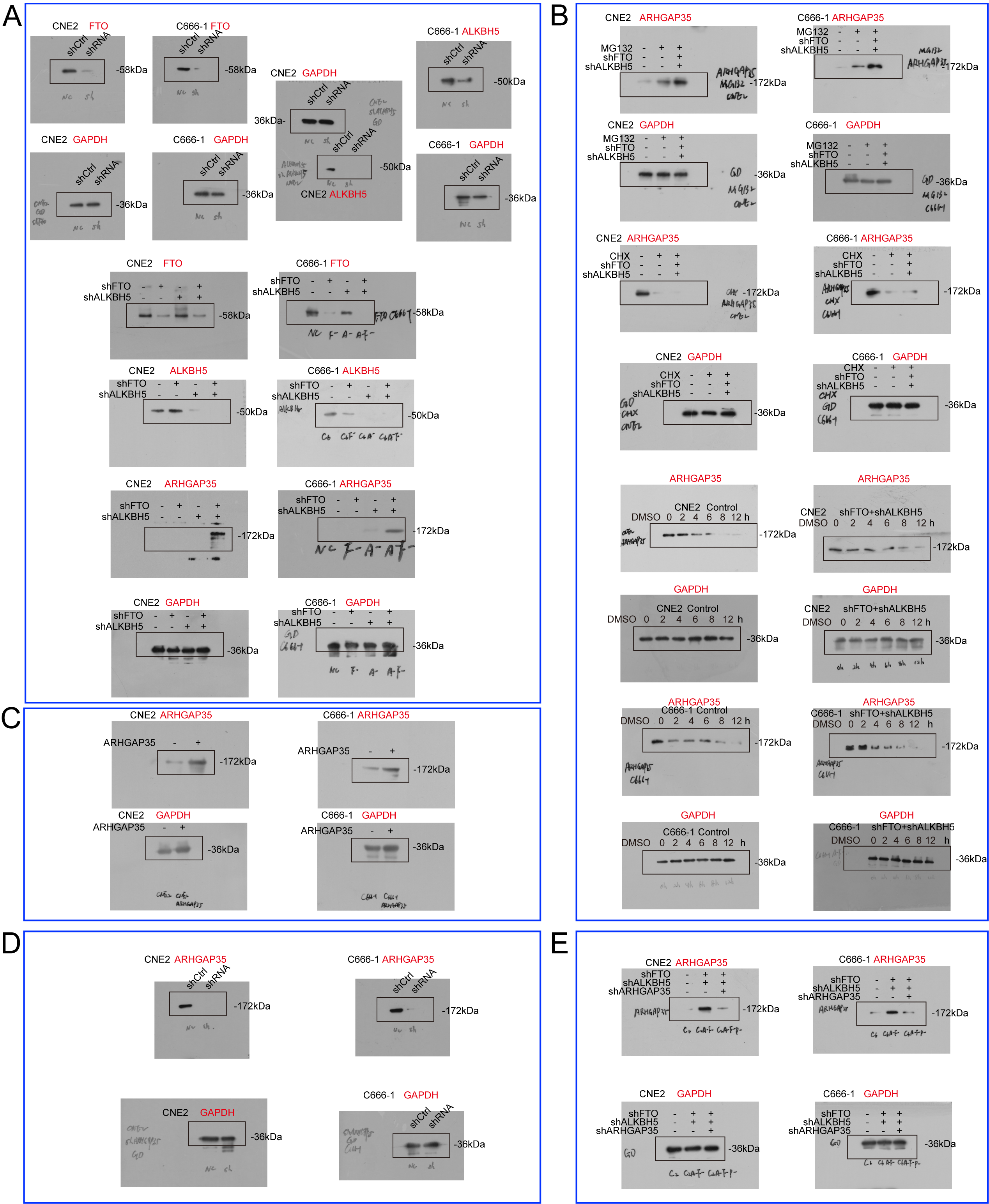

Supplement: Supplementary file 6 — Original Data File [file 41420_2024_1810_MOESM6_ESM.tif]
