# Supplementary material for: The m6A demethylases FTO and ALKBH5 aggravate the malignant progression of nasopharyngeal carcinoma by coregulating ARHGAP35
Source: Cell Death Discov. 2024 Jan 23;10:43. doi: 10.1038/s41420-024-01810-0 (PMC10806234; doi:10.1038/s41420-024-01810-0)
Supplement: Supplementary file 7 — Supply Table [file 41420_2024_1810_MOESM7_ESM.doc]

**Supplementary Tables**

**Supplementary Table S1. Clinicopathological parameters of NPC tissue microarrays in this study, related to Fig. 1 and Fig. 4.**

| No. | Survival status (Dead: 1; Alice: 0) | Survival time (Month) | Age | Metastasis (No: 0; metastasis: 1) | TNM clinical stage | Recurrence (No: 0; without-metastasis: 1; with-metastasis: 2) | Recurrence time | N stage |
| --- | --- | --- | --- | --- | --- | --- | --- | --- |
| 1 | 0 | 177 | 16 | 0 | 2 | 0 | - | 0 |
| 2 | 0 | 176 | 48 | 1 | 3 | 0 | - | 1 |
| 3 | 1 | 135 | 70 | 1 | 4 | 2 | 118 | 2 |
| 4 | 0 | 174 | 51 | 0 | 2 | 1 | 95 | 0 |
| 5 | 0 | 174 | 56 | 0 | 1 | 0 | - | 0 |
| 6 | 0 | 173 | 42 | 0 | 2 | 0 | - | 0 |
| 7 | 0 | 172 | 50 | 0 | 1 | 0 | - | 0 |
| 8 | 1 | 88 | 79 | 1 | 3 | 2 | 72 | 1 |
| 9 | 1 | 55 | 76 | 1 | 3 | 0 | - | 1 |
| 10 | 0 | 170 | 37 | 0 | 1 | 0 | - | 0 |
| 11 | 0 | 168 | 53 | 1 | 3 | 2 | 121 | 1 |
| 12 | 0 | 168 | 49 | 0 | 2 | 0 | - | 0 |
| 13 | 0 | 166 | 39 | 0 | 2 | 1 | 101 | 0 |
| 14 | 0 | 166 | 12 | 0 | 2 | 1 | 91 | 0 |
| 15 | 1 | 111 | 65 | 0 | 3 | 1 | 101 | 0 |
| 16 | 1 | 90 | 54 | 0 | 2 | 1 | 75 | 0 |
| 17 | 1 | 97 | 65 | 0 | 2 | 1 | 84 | 0 |
| 18 | 0 | 164 | 45 | 0 | 2 | 0 | - | 0 |
| 19 | 0 | 164 | 37 | 1 | 3 | 2 | 91 | 1 |
| 20 | 0 | 164 | 43 | 0 | 2 | 0 | - | 0 |
| 21 | 1 | 122 | 65 | 0 | 2 | 1 | 93 | 0 |
| 22 | 0 | 163 | 43 | 1 | 3 | 2 | 105 | 1 |
| 23 | 0 | 162 | 49 | 0 | 1 | 0 | - | 0 |
| 24 | 1 | 111 | 59 | 0 | 2 | 1 | 85 | 0 |
| 25 | 0 | 161 | 44 | 0 | 3 | 1 | 96 | 0 |
| 26 | 0 | 161 | 61 | 0 | 2 | 1 | 105 | 0 |
| 27 | 0 | 161 | 60 | 0 | 2 | 1 | 90 | 0 |
| 28 | 0 | 159 | 51 | 0 | 2 | 1 | 88 | 0 |
| 29 | 1 | 119 | 42 | 1 | 3 | 2 | 92 | 1 |
| 30 | 0 | 156 | 47 | 0 | 2 | 1 | 86 | 0 |
| 31 | 0 | 156 | 52 | 0 | 2 | 0 | - | 0 |
| 32 | 0 | 156 | 55 | 0 | 2 | 0 | - | 0 |
| 33 | 0 | 156 | 35 | 1 | 3 | 0 | - | 1 |
| 34 | 0 | 156 | 41 | 0 | 2 | 0 | - | 0 |
| 35 | 1 | 42 | 70 | 1 | 3 | 2 | 33 | 1 |
| 36 | 0 | 156 | 36 | 1 | 3 | 2 | 84 | 1 |
| 37 | 1 | 119 | 46 | 1 | 4 | 2 | 90 | 1 |
| 38 | 0 | 155 | 40 | 0 | 1 | 0 | - | 0 |
| 39 | 1 | 152 | 65 | 0 | 2 | 0 | - | 0 |
| 40 | 1 | 47 | 60 | 1 | 4 | 2 | 14 | 1 |
| 41 | 0 | 154 | 38 | 0 | 2 | 0 | - | 0 |
| 42 | 0 | 154 | 39 | 0 | 3 | 1 | 98 | 0 |
| 43 | 0 | 153 | 44 | 0 | 2 | 0 | - | 0 |
| 44 | 0 | 153 | 46 | 0 | 2 | 0 | - | 0 |
| 45 | 1 | 57 | 55 | 1 | 4 | 2 | 52 | 1 |
| 46 | 0 | 153 | 51 | 0 | 1 | 0 | - | 0 |
| 47 | 0 | 152 | 67 | 0 | 2 | 0 | - | 0 |
| 48 | 0 | 152 | 43 | 1 | 4 | 2 | 89 | 1 |
| 49 | 0 | 152 | 78 | 0 | 2 | 0 | - | 0 |
| 50 | 0 | 152 | 50 | 1 | 3 | 0 | - | 1 |
| 51 | 1 | 79 | 70 | 1 | 4 | 2 | 75 | 1 |
| 52 | 1 | 61 | 60 | 0 | 2 | 0 | - | 0 |
| 53 | 0 | 150 | 60 | 1 | 4 | 2 | 86 | 2 |
| 54 | 0 | 150 | 58 | 1 | 4 | 2 | 109 | 1 |
| 55 | 0 | 150 | 51 | 0 | 1 | 0 | - | 0 |
| 56 | 1 | 95 | 59 | 0 | 2 | 0 | - | 0 |
| 57 | 1 | 122 | 59 | 0 | 1 | 0 | - | 0 |
| 58 | 0 | 149 | 44 | 1 | 3 | 0 | - | 1 |
| 59 | 1 | 83 | 42 | 1 | 3 | 0 | - | 1 |
| 60 | 1 | 87 | 57 | 0 | 2 | 1 | 77 | 0 |
| 61 | 0 | 149 | 39 | 0 | 1 | 0 | - | 0 |
| 62 | 0 | 148 | 39 | 1 | 3 | 0 | - | 1 |
| 63 | 1 | 111 | 35 | 1 | 4 | 2 | 88 | 2 |
| 64 | 1 | 96 | 58 | 0 | 2 | 0 | - | 0 |
| 65 | 1 | 108 | 44 | 1 | 4 | 2 | 95 | 2 |
| 66 | 1 | 45 | 60 | 1 | 4 | 2 | 37 | 1 |
| 67 | 0 | 147 | 42 | 0 | 2 | 0 | - | 0 |
| 68 | 0 | 146 | 44 | 0 | 2 | 0 | - | 0 |
| 69 | 1 | 91 | 36 | 1 | 4 | 2 | 82 | 2 |
| 70 | 1 | 25 | 70 | 1 | 3 | 2 | 13 | 1 |
| 71 | 0 | 145 | 41 | 1 | 3 | 0 | - | 1 |
| 72 | 0 | 145 | 47 | 0 | 2 | 0 | - | 0 |
| 73 | 1 | 69 | 62 | 1 | 4 | 2 | 69 | 1 |
| 74 | 1 | 71 | 49 | 1 | 3 | 2 | 61 | 1 |
| 75 | 0 | 145 | 44 | 0 | 2 | 0 | - | 0 |
| 76 | 1 | 96 | 44 | 1 | 4 | 2 | 68 | 1 |
| 77 | 0 | 145 | 32 | 0 | 2 | 0 | - | 0 |
| 78 | 0 | 145 | 39 | 0 | 2 | 0 | - | 0 |
| 79 | 1 | 48 | 54 | 1 | 3 | 0 | - | 1 |
| 80 | 0 | 144 | 41 | 1 | 3 | 0 | - | 1 |
| 81 | 0 | 144 | 44 | 1 | 4 | 2 | 78 | 3 |
| 82 | 0 | 144 | 54 | 0 | 2 | 1 | 90 | 0 |
| 83 | 0 | 144 | 53 | 0 | 2 | 0 | - | 0 |
| 84 | 1 | 81 | 58 | 1 | 3 | 0 | - | 1 |
| 85 | 0 | 144 | 68 | 0 | 2 | 0 | - | 0 |
| 86 | 0 | 141 | 37 | 1 | 3 | 0 | - | 1 |
| 87 | 0 | 142 | 44 | 0 | 2 | 0 | - | 0 |
| 88 | 1 | 84 | 61 | 1 | 3 | 2 | 76 | 1 |
| 89 | 1 | 85 | 60 | 0 | 2 | 1 | 66 | 0 |
| 90 | 0 | 141 | 51 | 0 | 2 | 0 | - | 0 |
| 91 | 0 | 141 | 32 | 0 | 1 | 0 | - | 0 |
| 92 | 0 | 140 | 25 | 1 | 3 | 0 | - | 1 |
| 93 | 1 | 58 | 70 | 1 | 3 | 0 | - | 1 |
| 94 | 0 | 139 | 39 | 0 | 2 | 0 | - | 0 |
| 95 | 1 | 35 | 70 | 0 | 2 | 0 | - | 0 |
| 96 | 1 | 69 | 65 | 1 | 3 | 2 | 60 | 1 |
| 97 | 0 | 138 | 38 | 0 | 2 | 1 | 63 | 0 |
| 98 | 0 | 138 | 54 | 1 | 3 | 0 | - | 1 |
| 99 | 0 | 136 | 45 | 1 | 3 | 0 | - | 1 |
| 100 | 0 | 136 | 35 | 0 | 2 | 1 | 63 | 0 |
| 101 | 1 | 70 | 67 | 1 | 3 | 0 | - | 1 |
| 102 | 0 | 134 | 58 | 0 | 1 | 0 | - | 0 |
| 103 | 0 | 133 | 43 | 1 | 3 | 2 | 81 | 1 |
| 104 | 1 | 107 | 68 | 1 | 4 | 2 | 92 | 2 |
| 105 | 0 | 133 | 39 | 0 | 2 | 0 | - | 0 |
| 106 | 1 | 116 | 69 | 0 | 2 | 1 | 68 | 0 |
| 107 | 0 | 132 | 23 | 0 | 2 | 0 | - | 0 |
| 108 | 0 | 132 | 39 | 0 | 1 | 0 | - | 0 |
| 109 | 0 | 131 | 68 | 0 | 2 | 0 | - | 0 |
| 110 | 0 | 131 | 54 | 0 | 3 | 1 | 82 | 0 |
| 111 | 0 | 131 | 53 | 0 | 2 | 0 | - | 0 |
| 112 | 1 | 110 | 37 | 0 | 2 | 0 | - | 0 |
| 113 | 0 | 130 | 45 | 0 | 1 | 0 | - | 0 |
| 114 | 1 | 26 | 60 | 1 | 3 | 0 | - | 1 |
| 115 | 0 | 130 | 56 | 1 | 3 | 0 | - | 1 |
| 116 | 0 | 130 | 39 | 0 | 2 | 1 | 68 | 0 |
| 117 | 0 | 130 | 41 | 0 | 2 | 1 | 73 | 0 |
| 118 | 0 | 129 | 55 | 0 | 1 | 0 | - | 0 |
| 119 | 1 | 81 | 59 | 1 | 4 | 2 | 50 | 2 |
| 120 | 0 | 129 | 7 | 0 | 2 | 1 | 70 | 0 |
| 121 | 0 | 129 | 57 | 1 | 3 | 2 | 83 | 1 |
| 122 | 0 | 128 | 36 | 0 | 2 | 0 | - | 0 |
| 123 | 0 | 128 | 59 | 0 | 2 | 1 | 62 | 0 |
| 124 | 0 | 127 | 46 | 0 | 2 | 1 | 52 | 0 |
| 125 | 1 | 24 | 31 | 0 | 2 | 1 | 3 | 0 |
| 126 | 1 | 92 | 63 | 0 | 2 | 1 | 47 | 0 |
| 127 | 1 | 72 | 57 | 0 | 3 | 1 | 62 | 0 |
| 128 | 1 | 50 | 70 | 1 | 3 | 2 | 39 | 1 |
| 129 | 1 | 58 | 43 | 0 | 2 | 1 | 46 | 0 |
| 130 | 0 | 126 | 63 | 0 | 2 | 0 | - | 0 |
| 131 | 0 | 126 | 53 | 0 | 2 | 0 | - | 0 |
| 132 | 0 | 125 | 60 | 0 | 1 | 0 | - | 0 |
| 133 | 1 | 75 | 55 | 0 | 2 | 1 | 48 | 0 |
| 134 | 0 | 126 | 55 | 0 | 3 | 1 | 59 | 0 |
| 135 | 0 | 125 | 44 | 0 | 2 | 1 | 68 | 0 |
| 136 | 0 | 125 | 69 | 0 | 2 | 1 | 53 | 0 |
| 137 | 0 | 125 | 38 | 0 | 1 | 0 | - | 0 |
| 138 | 0 | 125 | 44 | 0 | 2 | 1 | 74 | 0 |
| 139 | 0 | 125 | 39 | 0 | 2 | 1 | 53 | 0 |
| 140 | 1 | 110 | 58 | 1 | 3 | 2 | 58 | 1 |
| 141 | 0 | 124 | 47 | 0 | 2 | 1 | 52 | 0 |
| 142 | 1 | 59 | 55 | 1 | 4 | 2 | 51 | 2 |
| 143 | 0 | 124 | 56 | 0 | 2 | 0 | - | 0 |
| 144 | 0 | 123 | 41 | 1 | 3 | 0 | - | 1 |
| 145 | 1 | 9 | 46 | 1 | 3 | 2 | 1 | 1 |
| 146 | 0 | 123 | 44 | 1 | 3 | 2 | 52 | 1 |
| 147 | 1 | 82 | 45 | 1 | 4 | 2 | 58 | 1 |
| 148 | 0 | 123 | 58 | 0 | 1 | 0 | - | 0 |
| 149 | 1 | 84 | 23 | 0 | 2 | 0 | - | 0 |
| 150 | 0 | 126 | 59 | 0 | 2 | 0 | - | 0 |
